# 2254. Asymptomatic Bacteriuria Underestimates True Inappropriate Prescribing for Non-Urinary Tract Infections

**DOI:** 10.1093/ofid/ofad500.1876

**Published:** 2023-11-27

**Authors:** Whitney Hartlage, Jeannie D Chan, Maria Bajenov, Alyssa Castillo, John B Lynch, Natalia Martinez-Paz, Chloe Bryson-Cahn, Zahra Escobar

**Affiliations:** VA Salt Lake City Medical Center, Salt Lake City, Utah; University of Washington, Seattle, Washington; University of Washington, Seattle, Washington; University of Colorado, Aurora, Colorado; Harborview Medical Center/U of Washington, Seattle, WA; University of Washington Tele-Antimicrobial Stewardship Program, Seattle, Washington; Harborview Medical Center, Seattle, Washington; University of Washington, Seattle, Washington

## Abstract

**Background:**

Unnecessary treatment of asymptomatic bacteriuria (ASB) is an important target for antimicrobial stewardship efforts. However, patients with urine colony counts <10^5^ colony-forming units (CFU)/mL or no growth are often excluded due to not meeting the definition of bacteriuria. We evaluated inappropriate treatment among patients without urinary tract infection (UTI) symptoms who underwent urine testing, regardless of the significance of growth in culture.

**Methods:**

Patients who underwent urine testing during September 8, 2022 - March 11, 2023, were reviewed across ten critical access hospitals participating in the University of Washington Center for Stewardship in Medicine. Culture growth was categorized as no growth, ≤10,000 CFU/mL, 11,000-50,000 CFU/mL, 51,000-100,000 CFU/mL or >100,000 CFU/mL. Asymptomatic for UTI was defined as the absence of documented signs or symptoms of UTI, per the National Hospital Safety Network definition and Infectious Diseases Society of America Guidelines. We assessed the number of patients with a positive UA (leukocyte esterase, WBC >10, or nitrites) treated with antibiotics and the association of urine colony cutoffs on inappropriate antibiotic prescribing. Variables were compared using Mantel-Haenszel chi square test. A two-sided p-value of < 0.05 was considered statistically significant.

**Results:**

The analysis included 616 patients- 249 were classified as asymptomatic for UTI (40%). Patients with asymptomatic pyuria (n=188) were more likely to receive treatment than those without pyuria (n=42) (79% vs 43%, p< 0.001). One hundred seventeen patients with urine colony counts < 10^5^ CFU/mL lacked signs or symptoms of UTI. Among these 117 patients, 72 (62%) were treated with antibiotics. There was a significant trend that treatment in asymptomatic patients increases with colony count in urine culture (p< 0.001).

**Figure:**
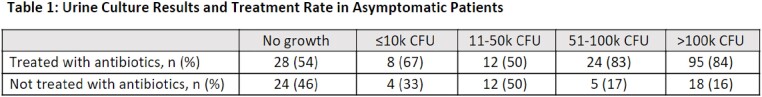

**Figure:**
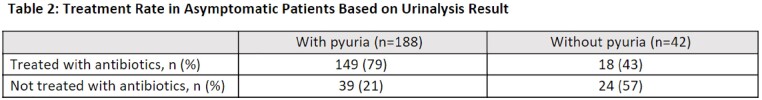

**Conclusion:**

Treatment of ASB alone inadequately captures the total number of patients receiving unnecessary antibiotics for suspected UTI. A more comprehensive means of quantifying unnecessary UTI treatment may be capturing all patients who undergo urine cultures-including those with asymptomatic pyuria or asymptomatic nitrituria, even with low growth on culture.

**Disclosures:**

**All Authors**: No reported disclosures

